# Immediate and long‐term effects of a very‐low‐calorie diet on diabetes remission and glycemic control in obese Thai patients with type 2 diabetes mellitus

**DOI:** 10.1002/fsn3.956

**Published:** 2019-02-11

**Authors:** Mongkontida Umphonsathien, Pornsawan Prutanopajai, Juntagan Aiam‐O‐Ran, Titiprang Thararoop, Apaporn Karin, Chanida Kanjanapha, Wiroj Jiamjarasrangsi, Weerapan Khovidhunkit

**Affiliations:** ^1^ Division of Endocrinology and Metabolism Department of Medicine Faculty of Medicine, and Hormonal and Metabolic Disorders Research Unit Chulalongkorn University and Excellence Center in Diabetes, Hormone and Metabolism King Chulalongkorn Memorial Hospital Thai Red Cross Society Bangkok Thailand; ^2^ Department of Dietetic and Diet Therapy King Chulalongkorn Memorial Hospital Thai Red Cross Society Bangkok Thailand; ^3^ Department of Preventive and Social Medicine Faculty of Medicine Chulalongkorn University Bangkok Thailand

**Keywords:** caloric restriction, obesity, quality of life, very‐low‐calorie diet

## Abstract

**Aim:**

A very‐low‐calorie diet (VLCD) can reverse the underlying defects of type 2 diabetes mellitus (DM) in obese subjects. We determined the efficacy, safety, and durability of VLCD in Thai patients with DM and obesity.

**Methods:**

Twenty Thai patients with DM and obesity were enrolled. After a 2‐week trial, VLCD (600 kcal/day) was continued for 8 weeks, followed by a 4‐week transition period. Data on diabetes remission (fasting plasma glucose level <126 mg/dl and HbA_1c_ <6.5% without the use of glucose‐lowering medications), glycemic control, metabolic parameters, and quality of life (QOL) were collected along with indices of insulin resistance (IR) and beta cell function. Glycemic control 12 months after discontinuation of VLCD was also examined.

**Results:**

Among 19 patients (age 48 ± 2 years, BMI 27.7 kg/m^2^) who completed the study, rapid improvement in glycemic control was observed in the first 2 weeks of VLCD. At both 8 and 12 weeks, diabetes remission was achieved in 79%. Significant weight loss was accompanied by a significant reduction in IR and an increase in beta cell function, starting at 4 weeks of VLCD. QOL also significantly increased. At 12 months after VLCD, however, DM remission was achieved in approximately 30%.

**Conclusion:**

Very‐low‐calorie diet was effective and safe in inducing short‐term diabetes remission in Thai subjects by ameliorating beta cell function and IR. Optimal long‐term glycemic control was potentially durable as one‐third of subjects remained without diabetes medication 12 months after VLCD.

## 1 INTRODUCTION

Type 2 diabetes mellitus (DM) is considered a growing pandemic with a rapid increase in the number of patients in both developed and developing countries (Jayawardena et al., 2012; Nanditha et al., 2016; Yang et al., 2016). A number of factors are thought to contribute to this pandemic, including a higher prevalence of obesity, population aging, and greater longevity due to advanced medical technologies. The global burden of DM and its complications is enormous, and effective measures to combat this disease on a large scale are clearly needed.

Type 2 DM is a chronic progressive disease with deterioration of beta cell function and beta cell mass over time. Multiple modalities of treatment have been widely used for patients with type 2 DM, but most of the available therapies fail to preserve beta cell function. Eventually, insulin therapy is frequently required to maintain acceptable glycemic control.

Recently, studies in obese patients undergoing gastric bypass surgery have demonstrated that metabolic abnormalities of type 2 DM can be reversed and diabetes medications can be discontinued soon after the operation (Nguyen & Varela, 2017). Normalization of plasma glucose concentrations occurs within the first week after the surgery, and it has been attributed to caloric restriction in the early postoperative period (Batterham & Cummings, 2016; Isbell et al., 2010; Lips et al., 2014). This finding has renewed interest in the role of caloric restriction in the management of obesity and type 2 DM.

Several studies have shown that improvement in glycemic control in patients with type 2 DM can be achieved by caloric restriction using a very‐low‐calorie diet (VLCD; Baker, Jerums, & Proietto, 2009; Capstick et al., 1997; Henry & Gumbiner, 1991; Lean et al., 2018; Malandrucco et al., 2012; Wing et al., 1994). In patients with severe obesity (body mass index [BMI] ~30 kg/m^2^), an improvement in hepatic insulin sensitivity was quickly observed only after a week after VLCD, whereas beta cell defect in insulin secretion was restored later at 8 weeks (Lim et al., 2011). In patients with extreme obesity (BMI ~45 kg/m^2^), however, only an improvement in insulin secretion was observed with no changes in hepatic insulin sensitivity (Malandrucco et al., 2012). The rate of diabetes remission appeared to be higher among those who lost more weight (Steven, Lim, & Taylor, 2013). Collectively, these studies suggest that the underlying pathophysiologic defects of type 2 DM can be reversed by VLCD (Lim et al., 2011; Malandrucco et al., 2012; Steven & Taylor, 2015; Steven et al., 2016). However, almost all of the VLCD studies have been performed in subjects of European descent who have high BMI. Asians with type 2 DM, in contrast, typically have lower BMI and exhibit more pronounced beta cell dysfunction compared to European counterparts (Ma & Chan, 2013). Data on caloric restriction in Asian patients with DM are extremely scarce, and no long‐term outcome has been reported. Whether VLCD in Asians would be equally effective as in Europeans and whether the pathophysiologic defects of type 2 DM can be reversed using VLCD similar to those in Europeans are currently unknown.

This study was designed to determine the efficacy, safety, and durability of a VLCD on the glycemic control and diabetes remission in Thai diabetes patients with obesity in an outpatient setting. Mechanistic insights into the improvement of glycemic control and quality of life of the patients after VLCD were also explored.

## 2 MATERIALS AND METHODS

### 2.1 Study design

The study design has been described in detail using CONSORT recommendation, and the study protocol was approved by our institutional research ethics committee. This clinical trial was registered at the Thai Clinical Trials Registry (TCTR20150930002 at http://www.clinicaltrials.in.th). Due to misunderstanding in the process of clinical trial registration, this trial was registered retrospectively and the ethics committee was immediately informed once this error was discovered.

### 2.2 Study population

Personnel of King Chulalongkorn Memorial Hospital with type 2 DM were recruited using advertisement and word of mouth. We chose to recruit our Hospital's personnel for this pilot study mainly because of safety and food logistics issues since all of them worked at the Hospital and most of them routinely ate their meals prepared by the Hospital on‐site. DM was defined as a fasting plasma glucose (FPG) level ≥126 mg/dl (7 mmol/L) or a 2‐hr glucose level after an oral glucose tolerance test (OGTT) ≥200 mg/dl (11.11 mmol/L) or the use of glucose‐lowering medication(s). The inclusion criteria at the time of screening were age between 20 and 60 years, glycated hemoglobin level (HbA_1C_) ≥6.5% (48 mmol/mol), DM duration <10 years, BMI 23–30 kg/m^2^, and good compliance to the study protocol. A BMI cutoff above 23 kg/m^2^ was chosen to represent being overweight according to the WHO criteria for Asians (WHO Expert Consultation, 2004). Exclusion criteria were previous treatment with a thiazolidinedione or a glucagon‐like peptide‐1 receptor agonist within the past 3 months, previous use of insulin, concurrent use of steroid, pregnancy, lactation, serum creatinine >1.5 mg/dl (0.08 mmol/L), serum alanine aminotransferase (ALT) >2.5‐fold above the upper limit of reference range, and fasting C‐peptide <1 ng/ml (331 pmol/L). All participants in this study gave written informed consent.

### 2.3 Study protocol

After the participants were screened for eligibility, they were followed in an outpatient setting. A detailed medical history, including comorbidities, medication use, health behaviors (smoking, alcohol use, and physical activity), and laboratory results, was obtained at screening visit, and routine physical examination was performed. Participants were advised to continue their habitual lifestyle pattern throughout the study.

**Figure 1:**
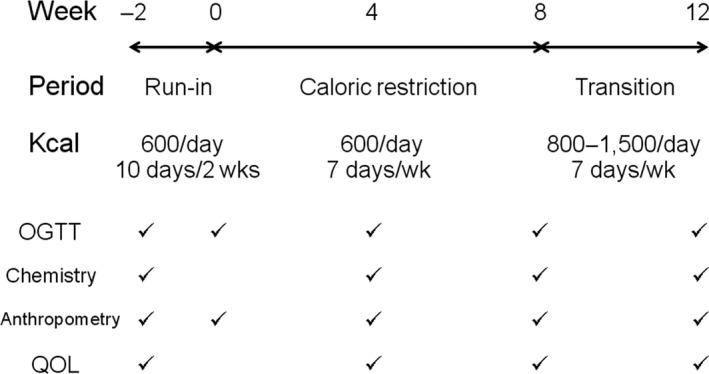
Study protocol. The study consisted of three periods. In the run‐in period (weeks −2 to 0), subjects were tried on a VLCD (600 kcal/day [2,512 KJ/day]) 10 days/2 weeks. In the caloric restriction period (weeks 0–8), subjects received a VLCD (600 kcal/day [2,512 KJ/day]) every day. In the transition period (weeks 8–12), subjects received higher caloric intake in a stepwise fashion from 800 kcal/day [3,349 KJ/day] on week 9 to 1,500 kcal/day [6,280 KJ/day] on week 12. An OGTT, blood chemistry, anthropometric measurements, and evaluation of QOL were assessed as indicated

The study protocol consists of three periods, a 2‐week run‐in period, an 8‐week caloric restriction period, and a 4‐week transition period (Figure 1). In the run‐in period, participants were tried on a VLCD (600 kcal/day [2,512 KJ/day]) for 10 days over a period of 2 weeks (weeks −2 to 0) to assess for compliance. Dietary record and urine ketone were used to monitor their compliance. Those who passed 90% rate of compliance were invited to continue on the caloric restriction period, during which participants received a VLCD for 8 weeks (weeks 0–8). After completing the caloric restriction period, the participants continued in the transition period for another 4 weeks (weeks 8–12). During this period, participants received higher caloric intake in a stepwise fashion (800 kcal/day [3,349 KJ/day] on week 9, 1,000 kcal/day [4,187 KJ/day] on week 10, 1,200 kcal/day [5,024 KJ/day] on week 11, and 1,500 kcal/day [6,280 KJ/day] on week 12). During all three study periods, patients were asked to monitor blood glucose levels by fingerstick at least twice weekly and when necessary if hypoglycemia was suspected. During the first several weeks, glucose‐lowering medications were adjusted by an endocrinologist based on fasting and postprandial glucose levels. In most cases, patients on oral hypoglycemic agent(s) had their medication(s) decreased or discontinued to avoid hypoglycemia. Weekly dietary recall and weekly urine ketone measurement were used to assess for dietary compliance. Participants were in close contact with an endocrinologist as well as a dietician using smartphones to maintain their glycemic control and compliance. All of them continued to work at the Hospital throughout the study periods, and for nurses and nursing assistants, they were advised to maintain their usual shift work since changes in rotational shift work have been associated with changes in glucose metabolism (Sharma et al., 2017). A 75‐g OGTT was performed, and blood samples were collected at weeks −2, 0, 4, 8, and 12 for assessment of glycemic and metabolic control.

The primary outcome was the rate of diabetes remission at the end of the study (weeks 8 and 12). Our definition of diabetes remission was based on the Consensus Statement (Buse et al., 2009), which defined remission as hyperglycemia not diagnostic of DM (FPG level <126 mg/dl [7 mmol/L] and HbA_1c_ <6.5% [48 mmol/mol]) in the absence of active pharmacologic therapy for DM. Because there is no consensus on the 2‐hr plasma glucose level after an OGTT to define diabetes remission (Buse et al., 2009), we only collected the data without using it in the definition of remission. Secondary outcomes were changes in glycemic control, safety parameters, insulin sensitivity and insulin secretion indices, anthropometric parameters, and quality of life. Safety parameters, including complete blood count, renal function, liver function test, and electrocardiogram (if necessary), were determined every 4 weeks. Anthropometric parameters and quality of life (QOL) were also assessed using an SF‐36 questionnaire at weeks −2, 4, 8, and 12.

After all patients finished the study protocol, they were sent back to their primary physicians for a regular follow‐up in an outpatient clinic. There was no intervention during this period, and data on glycemic control and use of glucose‐lowering medications were collected from the medical record up to 12 months after the protocol ended.

### 2.4 Calorie‐restricted diet protocol

A VLCD used in our study consisted of 54%–65% carbohydrate, 23%–30% protein, and 12%–13% fat with a total daily calorie of 600 kcal. The calories in our diet were divided evenly among the three meals. All meals during the study were prepared and provided by the Department of Dietetic and Diet Therapy of King Chulalongkorn Memorial Hospital using various selections of common Thai menus and recipes. We calculated dietary nutrient intake using the food composition tables of Nutrient calculation computer software (INMUCAL‐Nutrients V3 database NB1 2013) developed by Institute of Nutrition, Mahidol University, Nakorn Pathom, Thailand. Participants were required to pick up their meals daily. In case some of the participants were out of town, a 250‐ml can of Glucerna^®^ (Nestle, Amstelveen, the Netherlands) was provided to replace one meal. A list of non‐starchy vegetables was provided and allowed as a snack along with other energy‐free beverages. All subjects were encouraged to keep a daily dietary record and hand it in to the dietician at the end of the week. Subjects were encouraged to consume one tablet of multivitamins and a minimum of 2,500 ml of water daily.

### 2.5 Laboratory assays

Venous blood samples for measurement of glucose and insulin were drawn at 0, 30, 60, 90, and 120 min during an OGTT. Glucose and lipid levels were measured using enzymatic assay (Abbott Laboratories, Abbott Park, IL, USA). Low‐density lipoprotein (LDL) cholesterol level was calculated using the Friedewald formula. Plasma insulin level was assayed by solid‐phase enzyme‐labeled chemiluminescence immunometric assay (SIEMENS, Erlangen, Germany). C‐peptide level was assayed using a solid‐phase two‐site chemiluminescence immunoassay kit (SIEMENS) with an IMMULITE 1000 analyzer.

### 2.6 Insulin sensitivity and insulin secretion indices

Indices of insulin resistance (homeostasis model assessment‐insulin resistance or HOMA‐IR and quantitative insulin sensitivity check index or QUICKI) were calculated as previously described (Singh & Saxena, 2010). Briefly, HOMA1‐IR was calculated using the original equation [fasting plasma insulin (mU/L) × fasting plasma glucose (mmol/L)/22.5] while HOMA2‐IR was derived from the computer software (www.dtu.ox.ac.uk). Both indicate hepatic insulin resistance. QUICKI is a variation of HOMA‐IR by taking logarithmic and reciprocal transformation of the glucose‐insulin product [1/[log(I_0_) + log(G_0_)]: I_0_ = fasting plasma insulin (μU/ml), G_0_ = fasting plasma glucose (mg/dl)]. In addition, OGTT‐based measures of insulin sensitivity (Matsuda index) and insulin secretion (insulinogenic index) were also calculated. Matsuda index was derived to represent both hepatic and peripheral insulin sensitivities [10,000/√(fasting glucose × fasting insulin) (mean glucose × mean insulin)], whereas insulinogenic index indicates insulin response to a glucose challenge [∆insulin (0–30 min)/∆glucose (0–30 min)]. Lastly, oral disposition index, a composite measure of both insulin secretion and insulin sensitivity, was also determined [(1/fasting insulin) × (∆insulin (0–30 min)/∆glucose (0–30 min))] (Singh & Saxena, 2010).

### 2.7 Anthropometric parameters

Anthropometric parameters were assessed using a body composition analyzer (TANITIA model BC‐418) at weeks 0, 4, 8, and 12.

### 2.8 Quality of life

Quality of life (QOL) was also assessed using an SF‐36 questionnaire at weeks −2, 4, 8, and 12.

### 2.9 Statistical analyses

Power analysis was used to calculate the sample size based on data on diabetes remission by Lim, et al. (Lim et al., 2011). Assuming a 2‐sided significance level of 0.05, 18 subjects were needed to provide 90% of power to detect differences in an expected proportion of 0.95. The sample size of 20 participants allowed for a 10% dropout rate.

Statistical analyses were performed using SPSS 17.0 software. All data were presented as mean ± standard error of the mean (*SEM*) or median (interquartile range: IQR). A paired/unpaired Student's *t* test was used to compare data between the two groups. Analysis of variance (ANOVA) with repeated measures was used to detect changes in metabolic parameters over time during the study periods. Sidak correction was used for adjustment for multiple comparisons. Post hoc analysis was performed using the Bonferroni correction. Last‐observation‐carried‐forward (LOCF) imputation method was used for missing data. *p* value <0.05 was considered statistically significant.

## 3 RESULTS

### 3.1 Subject characteristics

**Figure 2:**
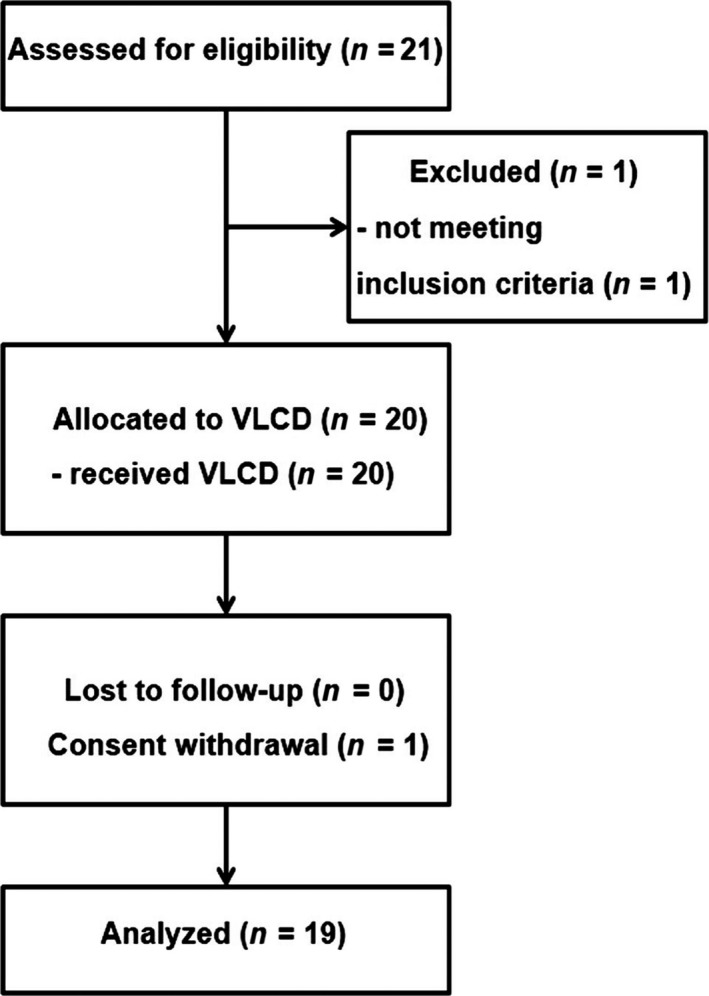
CONSORT flow diagram

A total of 21 patients with type 2 diabetes were recruited during January 2014–June 2014, and 1 was later excluded due to meeting the exclusion criteria. Twenty patients were enrolled in the study, but 1 withdrew consent during the run‐in period so the data were obtained for 19 patients (Figure 2). Baseline characteristics of the patients before the run‐in period are shown in Table 1 and Supporting Information Table S1. Because the majority of personnel in our Hospital were nursing staff, all but 1 were female. The mean age (±*SEM*) was 48 ± 1.7 years (range, 33–59), and the median duration of diabetes was 2.0 years (interquartile range: 0.4–8). History of glucose‐lowering medication use was as follows: sulfonylurea, metformin, and thiazolidinedione in 1, sulfonylurea, metformin, and alpha‐glucosidase inhibitor in 2, sulfonylurea and metformin in 4, metformin alone in 8, and no medications in 5.

**Table 1:** Baseline characteristics of the patients and effects of VLCD at various time points

	Week −2	Week 0		Week 4		Week 8		Week 12	
	Mean ± *SE*	Mean ± *SE*	*p*‐value^a^	Mean ± *SE*	*p*‐value^a^	Mean ± *SE*	*p*‐value^a^	Mean ± *SE*	*p*‐value^a^
FPG (mmol/L)	10.1 ± 0.9	7.0 ± 0.3	0.002	6.0 ± 0.4	0.001	5.2 ± 0.3	<0.001	6.3 ± 0.4	0.001
2‐hr postprandial glucose (mmol/L)	17.6 ± 1.8	12.9 ± 1.3	0.004	10.7 ± 0.9	0.011	9.9 ± 0.8	0.001	10.9 ± 0.7	0.006
HbA_1C_ (%)	8.0 ± 0.4	ND	–	6.8 ± 0.4	0.001	5.7 ± 0.2	<0.001	5.8 ± 0.1	0.001
HbA_1C_ (mmol/mol)	64 ± 5	ND	–	51 ± 4	0.001	38 ± 2	<0.001	40 ± 1	0.001
Total cholesterol (mmol/L)	5.1 ± 0.3	ND	–	4.8 ± 0.2	0.491	4.6 ± 0.2	0.084	5.7 ± 0.3	0.99
HDL cholesterol (mmol/L)	1.2 ± 0.1	ND	–	1.1 ± 0.1	0.99	1.1 ± 0.1	0.968	1.3 ± 0.1	0.99
Triglyceride (mmol/L)	2.0 ± 0.2	ND	–	1.0 ± 0.1	<0.001	0.9 ± 0.1	<0.001	1.2 ± 0.2	<0.001
LDL cholesterol (mmol/L)	3.0 ± 0.2	ND	–	3.1 ± 0.2	>0.99	3.1 ± 0.2	>0.99	3.7 ± 0.2	0.175
AST (U/L)	27 ± 4	ND	–	26 ± 2	0.99	24 ± 2	0.99	19 ± 1	0.317
ALT (U/L)	34 ± 5	ND	–	26 ± 3	0.895	23 ± 2	0.67	24 ± 4	0.99
Fasting insulin (μIU/ml)	13.8 ± 1.8	10.7 ± 2.0	0.80	6.7 ± 0.9	0.005	6.4 ± 0.8	0.004	7.2 ± 0.9	0.008
Fasting C‐peptide (ng/ml)	2.8 ± 0.2	2.4 ± 0.2	0.99	2.0 ± 0.2	0.009	1.5 ± 0.2	<0.001	1.8 ± 0.2	<0.001

*Notes*

ND: not done.

a Compared to values at week −2.

### 3.2 Plasma glucose response and diabetes remission

**Figure 3:**
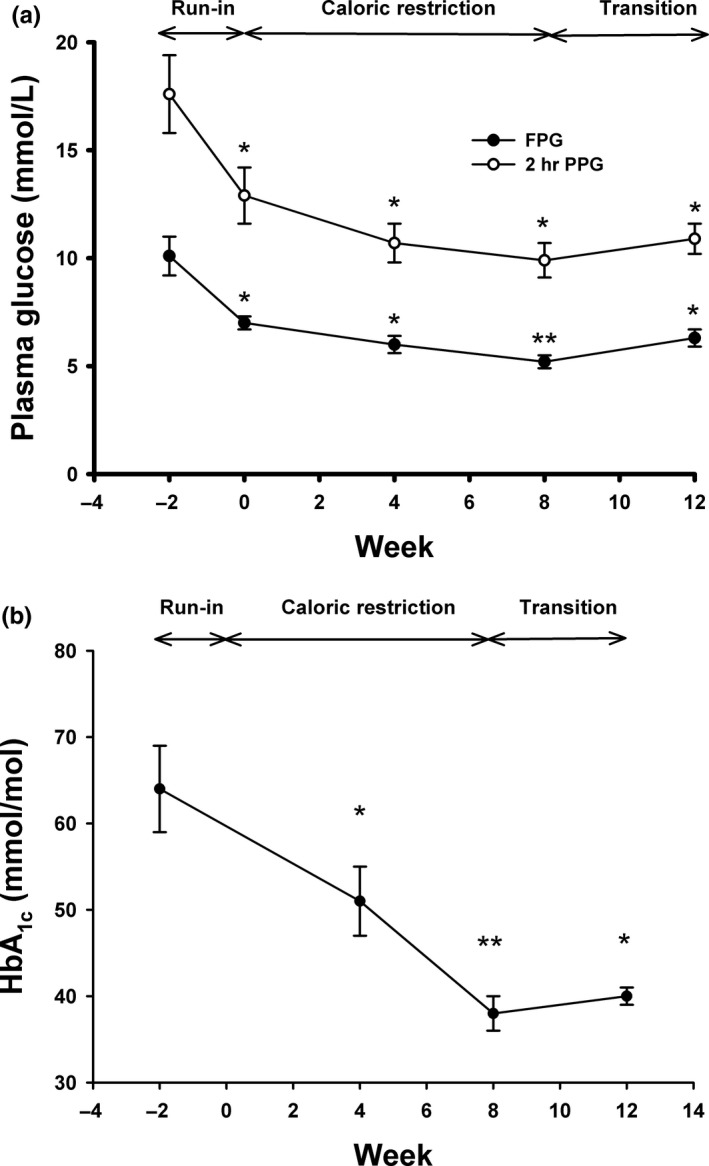
(a) Changes in fasting plasma glucose (FPG), 2‐hr plasma glucose after an OGTT (PPG), and (b) HbA_1c_ during the study periods. (a) Fasting plasma glucose (closed circles) and 2‐hr plasma glucose after an OGTT (open circles) were measured at weeks −2, 0, 4, 8, and 12. (b) HbA_1c_ was determined at weeks −2, 0, 4, 8, and 12. *: *p *<* *0.01, **: *p *<* *0.001 compared to values at week −2

During the run‐in period, plasma glucose started to decline in all subjects. At the end of the run‐in period (week 0), FPG levels were decreased by 57 mg/dl (3.2 mmol/L) on average (Figure 3a and Table 1). As a result, all diabetes medications were successfully withdrawn in each subject during this period to prevent hypoglycemia. Compliance to VLCD was considered good as evidenced by the dietary record and positive weekly urine ketone during the caloric restriction period; therefore, glycemic control continued to improve throughout. During the transitional period, the mean FPG levels increased slightly (Figure 3a and Table 1).

Similarly, after an OGTT, 2‐hr plasma glucose levels followed the similar pattern. The mean 2‐hr plasma glucose levels were significantly decreased at the end of the run‐in period and continued to decrease during the caloric restriction and the transition periods (Figure 3a and Table 1). As a result, HbA_1C_ levels were significantly decreased by 1.2% (13 mmol/mol), 2.3% (26 mmol/mol), and 2.2% (24 mmol/mol) at weeks 4, 8, and 12, respectively (Figure 3b).

**Figure 4:**
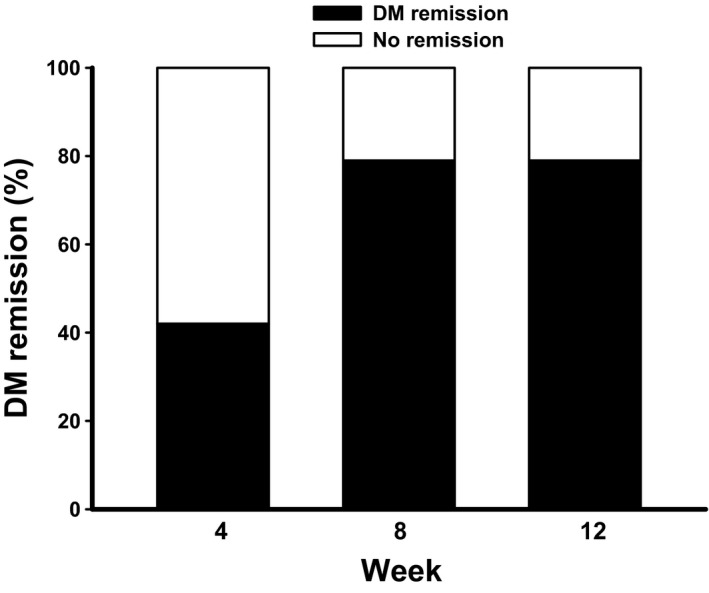
The percentage of remission of diabetes mellitus at different time points. Remission of diabetes mellitus, defined as a FPG level <126 mg/dl (7.0 mmol/L) and HbA_1c_ <6.5% (48 mmol/mol) without the use of glucose‐lowering medications, was determined at weeks 4, 8, and 12

At the end of the 8‐week caloric restriction period, diabetes remission, defined as a FPG level <126 mg/dl (7.0 mmol/L) and HbA_1c_ <6.5% (48 mmol/mol) without the use of glucose‐lowering medications, was achieved in 79% (15/19). Those who achieved diabetes remission at 8 weeks were slightly younger and had significantly higher fasting insulin and C‐peptide at baseline compared to those who did not achieve diabetes remission (Supporting Information Table S2). After the transition period, diabetes remission was still at 79% (15/19) (Figure 4). An intention‐to‐treat (ITT) analysis showed that diabetes remission was achieved in 75% at both 8 and 12 weeks.

### 3.3 Changes in metabolic parameters

Similar to the changes in glycemic control, we found that plasma triglyceride levels started to decrease early at week 4 of the caloric restriction period, reached the nadir at week 8, and increased slightly at week 12 (Table 1). However, serum levels of total cholesterol, HDL cholesterol, and LDL cholesterol did not significantly change throughout the study periods. Similarly, there were no significant changes in serum levels of AST and ALT at the end of the study (Table 1).

### 3.4 Changes in insulin sensitivity and insulin secretion indices

We next investigated whether the improvement in glycemic control was associated with a decrease in insulin resistance and/or an increase in beta cell function. We found that fasting plasma insulin levels started to decrease during the run‐in period but became significantly decreased at week 4 until week 12 (Table 1), which corresponded to the decrease in plasma glucose during these periods.

Markers of insulin resistance, HOMA1‐IR, HOMA2‐IR, and QUICKI, were significantly improved at week 4 until week 12 (Table 2). Matsuda index, which represents whole‐body insulin sensitivity, also increased significantly at week 4 until week 12. These results suggest that an improvement in glycemic control was associated with a reduction in insulin resistance and an improvement in whole‐body (both hepatic and peripheral) insulin sensitivity.

**Table 2:** Effect of VLCD on beta cell function, insulin sensitivity, and insulin resistance

	Week −2	Week 0		Week 4		Week 8		Week 12	
	Mean ± *SE*	Mean ± *SE*	*p*‐value	Mean ± *SE*	*p*‐value	Mean ± *SE*	*p*‐value	Mean ± *SE*	*p*‐value
HOMA1‐IR	5.8 ± 0.9	3.1 ± 0.5	0.053	1.8 ± 0.2	0.001	1.5 ± 0.2	0.001	1.9 ± 2.9	0.99
HOMA2‐IR	2.2 ± 0.3	1.4 ± 0.2	0.09	0.9 ± 0.1	<0.001	0.8 ± 0.1	0.001	0.9 ± 0.1	<0.001
QUICKI	0.31 ± 0.06	0.34 ± 0.10	0.11	0.36 ± 0.01	<0.001	0.38 ± 0.10	<0.001	0.36 ± 0.01	<0.001
Matsuda index	2.5 ± 0.3	4.6 ± 0.8	0.334	6.0 ± 0.5	<0.001	6.8 ± 0.9	0.012	5.6 ± 0.8	0.031
Insulinogenic index	0.2 ± 0.05	0.2 ± 0.07	0.99	0.3 ± 0.05	0.033	0.4 ± 0.08	0.016	0.3 ± 0.006	0.008
Disposition index	0.5 ± 0.1	1.2 ± 0.5	0.99	1.7 ± 0.3	0.005	2.3 ± 0.4	0.001	2.1 ± 0.5	0.042

Insulin secretion was assessed using an insulinogenic index, and it was found that there was a significant increase starting at week 4 and it persisted until week 12. Similarly, disposition index, which is a composite measure of beta cell function, also followed the similar pattern (Table 2). Collectively, these results suggest that the improvement in glycemic control during the caloric restriction period was associated with an improvement in both insulin sensitivity and beta cell function. The greatest improvement was seen at the end of week 8, which was the end of the caloric restriction period. During the transition period, there was a slight decline in the improvement of both insulin sensitivity and beta cell function. This pattern mirrors the changes in glycemic control during the same periods.

### 3.5 Changes in body weight, BMI, and body composition

A dramatic improvement in glycemic control, resulting in diabetes remission in the majority of patients, was accompanied by significant weight loss during the study. The amount of weight loss during these 14 weeks of dietary intervention was 9.5 ± 1.8 kg on average, which was equivalent to 13.3 ± 2.2% loss of an initial body weight (Table 3). The average amount of weight loss during the caloric restriction period was 1.0 kg/week. Not surprisingly, changes in BMI and waist circumference mirrored changes in body weight as shown in Table 3.

**Table 3:** Effect of VLCD on anthropometric measurement, body composition, and quality of life

	Week −2	Week 0		Week 4		Week 8		Week 12	
	Mean ± *SE*	Mean ± *SE*	*p*‐value	Mean ± *SE*	*p*‐value	Mean ± *SE*	*p*‐value	Mean ± *SE*	*p*‐value
Body weight (kg)	71.9 ± 2.9	69.2 ± 2.9	<0.001	65.6 ± 2.8	<0.001	61.3 ± 2.4	<0.001	62.4 ± 3.1	<0.001
BMI (kg/m^2^)	27.7 ± 0.8	26.1 ± 0.8	<0.001	24.7 ± 0.8	<0.001	24.0 ± 0.8	<0.001	23.7 ± 0.7	<0.001
Waist circumference (cm)	91.3 ± 2.0	89.7 ± 2.2	0.99	88.8 ± 2.1	0.077	84.7 ± 2.1	0.004	83.6 ± 2.2	<0.001
% Body fat	38.6 ± 1.3	37.3 ± 1.2	<0.001	34.7 ± 1.4	<0.001	32.8 ± 1.5	<0.001	32.2 ± 1.5	<0.001
Fat mass (kg)	27.0 ± 1.8	25.2 ± 1.6	<0.001	22.4 ± 1.6	<0.001	20.6 ± 1.6	<0.001	19.5 ± 1.5	<0.001
Fat‐free mass (kg)	41.6 ± 0.8	41.3 ± 0.8	0.093	40.9 ± 0.7	0.007	40.5 ± 0.8	<0.001	40.5 ± 0.7	0.004
Muscle mass (kg)	39.2 ± 0.7	38.8 ± 0.7	0.096	38.5 ± 0.7	0.011	38.0 ± 0.7	<0.001	38.1 ± 0.7	0.003
TBW (kg)	45.5 ± 0.9	45.2 ± 1.2	0.071	47.9 ± 0.9	0.001	49.0 ± 0.9	<0.001	50.0 ± 0.9	<0.001
Quality of life	2,494 ± 161	ND	–	2,695 ± 134	0.13	2,880 ± 111	0.006	3,040 ± 100	<0.001

*Note*

ND: not done.

Following caloric restriction, there was a marked reduction in both percentages of body fat and fat mass (Table 3). Fat mass progressively and significantly decreased by 28% from week −2 to week 12. Fat‐free mass and muscle mass also significantly decreased, but to a less extent, representing only a 2.6% and a 2.8% reduction, respectively (Table 3).

### 3.6 Quality of life (QOL)

Quality of life, assessed using an SF‐36 questionnaire, showed a significant increase in the scores at weeks 8 and 12 (Supporting Information Table S3), mainly in the physical function, general health, and health change domains, which suggested an improvement in health‐related QOL.

### 3.7 Side effects

No serious side effects were observed. Five patients experienced constipation, and three patients reported numbness. Paresthesia was improved after taking a multivitamin daily. Generalized hair loss and transient hearing impairment were reported in 2 and 1 patients, respectively. There were no changes in serum creatinine level throughout the study periods. No evidence of cardiac arrhythmias was observed.

### 3.8 Long‐term glycemic effects

After all subjects completed the study, all but 2 who resigned from the Hospital (*n* = 17) were followed in an outpatient clinic with their physicians. Data on glycemic control were collected, and none of them had a repeat OGTT. The mean FPG and HbA_1c_ were 150 ± 16 mg/dl (8.3 ± 0.9 m/L) and 7.3 ± 0.4% (56 ± 4 mmol/mol) at 6 months and 153 ± 13 mg/dl (8.5 ± 0.7 mmol/L) and 7.5 ± 0.4% (58 ± 4 mmol/mol) at 12 months after VLCD, respectively. Using FPG and HbA_1c_ cutoffs, diabetes remission was 35% at 6 months (30% by ITT analysis) and 24% at 12 months (20% by ITT analysis). Data on body weight and BMI, however, were unavailable. Interestingly, no significant differences in clinical characteristics were found between those who achieved and those did not achieve remission of diabetes at 12 months (Supporting Information Table S4).

## 4 DISCUSSION

Our study has demonstrated that caloric restriction by means of VLCD in obese Thai patients with type 2 DM can be executed effectively and safely in an outpatient setting. A rapid improvement in glycemic control led to discontinuation of glucose‐lowering medications in all patients in the first 2 weeks. Optimal glycemic control continued to sustain during the course of VLCD, and after 8 weeks of VLCD, almost 80% of patients were in diabetes remission. We found that the improvement in glycemic control was associated with an improvement in both insulin sensitivity and beta cell function. Significant weight loss and significant improvement in QOL were also observed. Improvement in glycemic control was, however, short‐lived and, without further intervention, only approximately a quarter of subjects remained in diabetes remission.

Very‐low‐calorie diet has long been studied in obese subjects since the late 1950s (Atkinson et al., 1993; Wadden, Stunkard, & Brownell, 1983). Although multiple metabolic benefits have been observed, controversies surrounding VLCD still exist, especially regarding its safety (Henry & Gumbiner, 1991; Wadden et al., 1983). In addition, long‐term compliance is a critical issue, which limits its use in obese subjects, including those with type 2 DM (Wing, 1995). Recent interest in VLCD, however, has been renewed (Baker et al., 2009; Manco & Mingrone, 2005; Soare, Weiss, & Pozzilli, 2014) with a pandemic of obesity and a hypothesis linking caloric restriction to remission of type 2 DM following a bariatric surgery (Isbell et al., 2010; Jackness et al., 2013; Lips et al., 2014). Use of VLCD, one form of caloric restriction, has recently been examined in obese subjects with type 2 DM (Lim et al., 2011; Malandrucco et al., 2012; Steven & Taylor, 2015; Steven et al., 2013, 2016). Rapid improvement in glycemic control leading to short‐term diabetes remission is observed.

Type 2 DM is a heterogeneous disorder, and certain differences have been observed among Asian and European patients (Ma & Chan, 2013). For example, type 2 DM often develops in Asian patients at the lower BMI than that of European patients. At any given BMI, Asian patients tend to have more visceral adiposity than those European counterparts. Furthermore, early beta cell dysfunction has been noted in Asian patients, resulting in development of DM at a younger age. Even among Asian patients, in fact, differences in ethnic‐specific diabetes phenotypes also exist between East Asians and South Asians (Maskarinec et al., 2009, 2015).

Data on caloric restriction in Asian patients with obesity and type 2 DM, however, are relatively scarce, and its long‐term durability is unknown. With increasing prevalence of obesity and type 2 DM in Asian countries (Jayawardena et al., 2012; Nanditha et al., 2016; Yang et al., 2016), it is therefore critically important to investigate the effects of VLCD in this specific population. To our knowledge, only one preliminary report of a very short‐term VLCD in obese Chinese subjects with type 2 DM had been published (Liu et al., 2015). Nine days of VLCD resulted in an improvement in glycemic control and a reduction in insulin resistance. Our study, using VLCD for a prolonged period of time, further showed this modality of treatment is effective and safe in Asian patients with lower BMI than that of Europeans (~27.7 kg/m^2^ in our study). It is of note that the magnitude of HbA_1c_ reduction in our study at the end of 8 weeks was 2.3% (26 mmol/mol), which appears to be similar to that of insulin or a combination of oral hypoglycemic agents, but without need for medication administration. Improvements in insulin sensitivity, beta cell function, and quality of life were also demonstrated. Although we believe that improvement in glucose metabolism in this study was mainly the effect of VLCD, we cannot rule out the possibilities that other confounding factors might contribute to this improvement. For example, introduction of self‐blood glucose monitoring has been associated with a modest improvement in glycemic control (Zhu, Zhu, & Leung, 2016) and an increase in water intake, which, we encouraged during VLCD, might also be associated with a reduction in body weight (Muckelbauer, Sarganas, Gruneis, & Muller‐Nordhorn, 2013).

Published in 2009, the Consensus Statement of diabetes remission defined remission as partial and complete (Buse et al., 2009). While partial remission was defined as hyperglycemia below diagnostic criteria of DM (FPG 100–126 mg/dl [5.6–7.0 mmol/mol] and HbA_1c_ <6.5% [48 mmol/mol]) for at least 1 year without use of glucose‐lowering medications, criteria for complete remission were stricter with FPG <100 mg/dl and normal HbA_1c_. The criteria used in our study are, therefore, almost consistent with partial remission according to the Consensus Statement (Buse et al., 2009). So far, only a few studies reported a long‐term effect of VLCD on glycemic control and diabetes remission (Lean et al., 2018; Paisey et al., 2002; Steven et al., 2016). Our current study also provides a crucial piece of information on the durability effect of VLCD in Asian patients. Similar to other studies in patients of European descent, the beneficial effect on glycemic control after discontinuation of VLCD is potentially durable only in a minority of patients. The results of the Look AHEAD study, which implemented a low‐calorie‐diet (1,200–1,800 kcal/day [5,024.2 ± 7,536.2 KJ]) and moderate‐intensity physical activity, also showed that the long‐term remission rate of diabetes was modest (Gregg et al., 2012). Collectively, these pieces of information suggest that continued interventions and/or reinforcements are necessarily required after discontinuation of caloric restriction in order to maintain acceptable glycemic control in the long term. A recently reported DiRECT study performed in primary care in Scotland and England has supported this concept. Although using only a HbA_1c_ criterion <6.5% (48 mmol/L) for defining diabetes remission, a remarkable remission rate of 46% at 1 year was found (Lean et al., 2018; Taylor et al., 2018). It is of note that the duration of VLCD in the DiRECT study was longer (3–5 months) than that of our study and structured support for long‐term weight control was readily implemented after discontinuation of VLCD.

The limitations of this study must be considered. First, the sample size of the study was small since we initially designed this study to test the possibility of VLCD prepared in‐house by our Hospital in an outpatient setting. In addition, due to the nature of our Hospital personnel, mainly nurses and nursing assistants, almost all of the studied subjects were female, thus limiting the generalizability to the male subjects. A plan for more subjects, longer duration, and effective ways to maintain VLCD in the long term is currently underway. Second, there was no control group in our study. However, previous studies have repeatedly shown that it was highly unlikely for the control group to achieve rapid glycemic control and diabetes remission as demonstrated by VLCD (Gregg et al., 2012; Lim et al., 2011). Thirdly, the participants were selected to include only non‐insulin‐treated patients; therefore, effects in those using insulin are unknown. Lastly, insulin sensitivity indices in our study were obtained from OGTT, not from the gold‐standard euglycemic hyperinsulinemic clamp, but our results were consistent with other previous studies in other populations (Jackness et al., 2013; Lim et al., 2011; Malandrucco et al., 2012; Steven et al., 2016).

In conclusion, our results show that VLCD was effective and safe in improving short‐term glycemic control in obese Thai subjects with type 2 DM, resulting in diabetes remission in a majority of patients. Given a pandemic of obesity and type 2 DM in Asia, this modality of treatment may be an interesting option for certain patients, but, without further intervention after discontinuation of VLCD, glycemic durability of VLCD is possible only in a minority of subjects.

## CONFLICT OF INTEREST

JA, AK, CK, and WJ declare that they have no conflict of interest. WK received lecture fees from Astra Zeneca, Sanofi Aventis, Novo Nordisk, and Biopharm. MU, PP, TT, and WK coauthored a Thai pocketbook with copyrights on low‐calorie menus.

## ETHICAL STATEMENT

The study conforms to the Declaration of Helsinki Guidelines for human subjects. All participants in the study gave written informed consents. The study protocol was ethically reviewed and approved by our Institutional Research Ethics committee (Chulalongkorn University).

## TRANSPARENCY STATEMENT

The lead author affirms that this manuscript is an honest accurate and transparent account of the study being reported. The reporting of this work is compliant with CONSORT guideline. The lead author affirms that no important aspects of the study have been omitted and that any discrepancies from the study as planned have been explained.
