# Supplementary material for: Immediate and long‐term effects of a very‐low‐calorie diet on diabetes remission and glycemic control in obese Thai patients with type 2 diabetes mellitus
Source: Food Sci Nutr. 2019 Feb 11;7(3):1113–22. doi: 10.1002/fsn3.956 (PMC6418422; doi:10.1002/fsn3.956)
Supplement: Supplementary file 1 [file FSN3-7-1113-s001.docx]

**Supporting information**

**S1 Table. Baseline characteristics**

**S2 Table. Clinical characteristics of patients who achieved and did not achieve remission of diabetes mellitus at 8 weeks**

**S3 Table. SF-36 Quality of life scores at 0, 4, 8 and 12 weeks**

**S4 Table. Clinical characteristics of patients who achieved and did not achieve remission of diabetes mellitus at 12 months**

**S1 Table. Baseline characteristics of the patients before the run-in period**

| Variable |  |
| --- | --- |
| Age (yr) | 48.2 ± 1.7 |
| Female sex – no. (%) | 18 (95%) |
| Mean duration of diabetes (yr) | 2.0 (0.4 - 8.0) |
| Hypertension – no. (%) | 8 (42%) |
| Dyslipidemia – no. (%) | 12 (63%) |
| Smoker – no. (%) | 0 (0%) |
| Body weight (kg) | 71.9 ± 2.9 |
| Body mass index (kg/m^2^) | 27.7 ± 0.8 |
| Waist circumference (cm) | 91.3 ± 2.0 |
| HbA_1c_ (%) | 8.0 ± 0.4 |
| HbA_1c_ (mmol/mol) | 64 ± 5 |
| Total cholesterol (mmol/L) | 5.1 ± 0.3 |
| HDL cholesterol (mmol/L) | 1.2 ± 0.1 |
| Triglyceride (mmol/L) | 2.0 ± 0.2 |
| LDL cholesterol (mmol/L) | 3.0 ± 0.2 |
| AST (U/L) | 27 ± 4 |
| ALT (U/L) | 34 ± 5 |

Data are presented as mean ± SEM or median (interquartile range)

**S2 Table. Clinical characteristics of patients who achieved and did not achieve remission of diabetes mellitus at 8 weeks**

| Variable | Diabetes remission  (n=15) | No diabetes remission  (n=4) | P value |
| --- | --- | --- | --- |
| Age (yr) | 46.7 ± 1.9 | 54.0 ± 2.0 | 0.03 |
| Mean duration of diabetes (yr) | 2.0 (1.0 – 5.0) | 6.0 (2.8 – 8.5) | 0.12 |
| Body weight (kg) | 70.7 ± 2.7 | 76.7 ± 10.2 | 0.60 |
| Body mass index (kg/m^2^) | 27.8 ± 0.8 | 28.2 ± 1.2 | 0.82 |
| Waist circumference (cm) | 90.4 ± 1.9 | 95.0 ± 7.0 | 0.56 |
| FPG (mmol/L) | 9.1 ± 0.7 | 14.0 ± 3.2 | 0.23 |
| HbA_1c_ (%) | 7.5 ± 0.4 | 9.8 ± 0.8 | 0.05 |
| Total cholesterol (mmol/L) | 5.0 ± 0.2 | 5.6 ± 1.0 | 0.63 |
| HDL cholesterol (mmol/L) | 1.2 ± 0.1 | 1.3 ± 0.2 | 0.47 |
| Triglyceride (mmol/L) | 2.0 ± 0.2 | 2.0 ± 0.6 | 0.97 |
| LDL cholesterol (mmol/L) | 2.9 ± 0.2 | 3.3 ± 0.8 | 0.68 |
| AST (U/L) | 29 ± 4 | 18 ± 3 | 0.06 |
| ALT (U/L) | 39 ± 6 | 18 ± 6 | 0.03 |
| Fasting insulin (µIU/mL) | 15.7 ± 2.0 | 6.7 ± 1.7 | 0.005 |
| Fasting C-peptide (ng/mL) | 2.9 ± 0.2 | 2.3 ± 0.05 | 0.02 |
| Percent of weight loss at 8 weeks (%) | 13.5 ± 1.3 | 11.9 ± 4.2 | 0.74 |

Data are presented as mean ± SEM or median (interquartile range)

**S3 Table. SF36 Quality of life scores at 0, 4, 8, and 12 weeks**

| **Domain** | **Week** | **Mean ± SE** | **Mean difference ± SE** | **P-value** |
| --- | --- | --- | --- | --- |
| **Physical function** | -2 | 738.4 ± 45.0 | - | - |
|  | 4 | 844.7 ± 22.3 | 76.3 ± 40.5 | 0.076 |
|  | 8 | 881.6 ± 24.2 | 113.5 ± 40.4 | 0.012 |
|  | 12 | 889.5 ± 25.8 | 121.1 ± 38.5 | 0.006 |
|  | | | | |
| **Role limitation due to physical health** | -2 | 263.2 ± 39.2 | - | - |
|  | 4 | 278.9 ± 37.9 | 15.8 ± 34.4 | 0.652 |
|  | 8 | 342.1 ± 23.3 | 78.9 ± 32.1 | 0.024 |
|  | 12 | 373.7 ± 15.0 | 110.5 ± 36.6 | 0.007 |
|  | | | | |
| **Role limitation due to emotional problems** | -2 | 236.8 ± 21.9 | - | - |
|  | 4 | 233.7 ± 24.2 | -3.2 ± 29.0 | 0.914 |
|  | 8 | 276.8 ± 9.6 | 40.0 ± 22.1 | 0.087 |
|  | 12 | 266.3 ± 18.7 | 29.5 ± 18.8 | 0.135 |
|  | | | | |
| **Energy/fatigue** | -2 | 265.3 ± 17.5 | - | - |
|  | 4 | 257.9 ± 15.5 | -7.4 ± 15.9 | 0.649 |
|  | 8 | 276.8 ± 16.1 | 11.6 ± 16.6 | 0.495 |
|  | 12 | 301.1 ±18.4 | 35.8 ± 13.5 | 0.016 |
|  | | | | |
| **Emotional well-being** | -2 | 380.0 ± 22.4 | - | - |
|  | 4 | 374.7 ± 23.5 | -5.3 ± 15.9 | 0.744 |
|  | 8 | 402.1 ± 15.4 | 22.1 ± 20.1 | 0.285 |
|  | 12 | 414.7 ± 21.0 | 34.7 ± 14.7 | 0.029 |
|  | | | | |
| **Social function** | -2 | 152.6 ± 11.0 | - | - |
|  | 4 | 150.0 ± 9.6 | -2.6 ± 10.5 | 0.904 |
|  | 8 | 156.6 ± 9.7 | 3.9 ± 16.9 | 0.818 |
|  | 12 | 168.4 ± 6.9 | 15.7 ± 12.0 | 0.204 |
|  | | | | |
| **Pain** | -2 | 142.6 ± 10.5 | - | - |
|  | 4 | 149.2 ± 9.4 | 5.5 ± 8.7 | 0.535 |
|  | 8 | 160.8 ± 10.0 | 18.2 ± 14.6 | 0.229 |
|  | 12 | 149.2 ± 9.4 | 6.6 ± 8.4 | 0.446 |
|  | | | | |
| **General health** | -2 | 269.2 ± 24.5 | - | - |
|  | 4 | 322.4 ± 26.5 | 53.2 ± 24.9 | 0.047 |
|  | 8 | 360.5 ± 25.2 | 91.3 ± 25.8 | 0.002 |
|  | 12 | 385.5 ± 21.1 | 116.3 ± 21.2 | <0.001 |
|  | | | | |
| **Health change** | -2 | 48.7 ± 5.7 | - | - |
|  | 4 | 71.1 ± 6.4 | 22.4 ± 8.9 | 0.022 |
|  | 8 | 81.6 ± 5.7 | 32.9 ± 8.6 | 0.001 |
|  | 12 | 93.4 ± 2.6 | 44.7 ± 5.3 | <0.001 |

**S4 Table. Clinical characteristics of patients who achieved and did not achieve remission of diabetes mellitus at 12 months**

| Variable | Diabetes remission  (n=4) | No diabetes remission  (n=13) | P value |
| --- | --- | --- | --- |
| Age (yr) | 47.8 ± 5.1 | 47.8 ± 2.0 | 0.99 |
| Mean duration of diabetes (yr) | 6.0 (1.6 – 7.8) | 2.0 (1.1 – 4.5) | 0.25 |
| Body weight (kg) | 78.6 ± 10.1 | 69.1 ± 2.8 | 0.43 |
| Body mass index (kg/m^2^) | 29.4 ± 1.5 | 27.4 ± 0.9 | 0.32 |
| Waist circumference (cm) | 98.5 ± 5.5 | 89.3 ± 2.1 | 0.20 |
| FPG (mmol/L) | 11.6 ± 0.7 | 9.0 ± 0.7 | 0.03 |
| HbA_1c_ (%) | 9.1 ± 0.5 | 7.5 ± 0.4 | 0.06 |
| Total cholesterol (mmol/L) | 4.5 ± 0.6 | 5.0 ± 0.1 | 0.49 |
| HDL cholesterol (mmol/L) | 1.4 ± 0.2 | 1.2 ± 0.1 | 0.46 |
| Triglyceride (mmol/L) | 1.9 ± 0.4 | 1.9 ± 0.2 | 0.90 |
| LDL cholesterol (mmol/L) | 2.4 ± 0.6 | 3.0 ± 0.2 | 0.42 |
| AST (U/L) | 28 ± 8 | 28 ± 5 | 0.93 |
| ALT (U/L) | 26 ± 9 | 40 ± 7 | 0.23 |
| Fasting insulin (µIU/mL) | 13.0 ± 4.7 | 14.8 ± 2.2 | 0.74 |
| Fasting C-peptide (ng/mL) | 3.3 ± 0.8 | 2.6 ± 0.2 | 0.48 |
| Percent of weight loss at 8 weeks (%) | 14.6 ± 2.8 | 13.7 ± 1.3 | 0.78 |
| Percent of weight loss at 12 weeks (%) | 13.2 ± 2.7 | 15.0 ± 2.0 | 0.61 |

Data are presented as mean ± SEM or median (interquartile range)
